# Mitochondrial genomes and phylogenomic analysis of *Plectostylus broderipii* and *Bostryx erythrostoma* (Gastropoda, Stylommatophora, Orthalicoidea) from the Atacama Desert, Chile

**DOI:** 10.3897/zookeys.1291.197983

**Published:** 2026-09-01

**Authors:** Roberto Contreras-Díaz, Rodrigo M. Barahona-Segovia, Ricardo Catalán-Garrido, Juan Francisco Araya, Mariana Arias-Aburto, César Pizarro-Gacitúa

**Affiliations:** 1 Centro Regional de Investigación y Desarrollo Sustentable de Atacama (CRIDESAT), Universidad de Atacama, Copiapó, Chile Centro Regional de Investigación y Desarrollo Sustentable de Atacama (CRIDESAT), Universidad de Atacama Copiapó Chile; 2 Laboratorio de Ecología y Conservación de Invertebrados (LECI), Departamento de Ciencias Biológicas y Biodiversidad, Universidad de Los Lagos, Osorno, Chile SEREMI del Medio Ambiente Región de Atacama, Ministerio de Medio Ambiente, Gobierno de Chile Copiapó Chile; 3 SEREMI del Medio Ambiente Región de Atacama, Ministerio de Medio Ambiente, Gobierno de Chile, Copiapó, Chile Departamento de Zoología, Facultad de Ciencias Naturales y Oceanográficas, Universidad de Concepción Concepción Chile; 4 Departamento de Zoología, Facultad de Ciencias Naturales y Oceanográficas, Universidad de Concepción, Concepción, Chile Laboratorio de Ecología y Conservación de Invertebrados (LECI), Departamento de Ciencias Biológicas y Biodiversidad, Universidad de Los Lagos Osorno Chile; 5 Corporación Nacional Forestal (CONAF), Región de Atacama, Copiapó, Chile Corporación Nacional Forestal (CONAF), Región de Atacama Copiapó Chile

**Keywords:** Atacama Desert, Bothriembryontidae, Bulimulidae, land snails, mitogenome annotation, mitogenomics, next-generation sequencing

## Abstract

We report the first mitochondrial genomes of two Orthalicoidea land snails from the Atacama Desert of northern Chile, *Bostryx erythrostoma* and *Plectostylus broderipii*. Using Illumina short-read sequencing, we assembled and annotated mitochondrial genomes of 15,525 bp and 14,978 bp, respectively, with strong A+T bias (69.75% in *B. erythrostoma*; 72.08% in *P. broderipii*). Both genomes retain the standard metazoan mitochondrial complement of 37 genes, including 13 protein-coding genes (PCGs), 22 tRNAs, and two rRNAs, with differences in total length mainly due to variation in non-coding regions rather than coding-gene content. Comparative analysis showed that the two species share the same order of the 13 PCGs, whereas differences are concentrated in local tRNA/rRNA rearrangements and intergenic architecture. Both mitogenomes also exhibit negative AT skew and positive GC skew under a standardized orientation. Phylogenetic inference based on a concatenated 13-PCG nucleotide supermatrix resolved both taxa within Orthalicoidea with strong support: *B. erythrostoma* grouped with a congeneric *Bostryx* reference, and this clade clustered with *Rabdotus mooreanus* and *Naesiotus nux*, whereas *P. broderipii* was inferred as sister to this orthalicoid cluster.

## Introduction

The land-snail fauna of northern Chile remains unevenly documented, with many taxa still poorly known beyond shell-based descriptions and sparse locality records (Araya 2015). This knowledge gap is particularly evident in the coastal desert of the Atacama Region, an arid to hyper-arid landscape where terrestrial molluscs often occur in patchy distributions and are seldom observed alive (Araya and Catalán 2014). Within this broader context, *Bostryx* Troschel, 1847 stands out as one of the most diverse and taxonomically challenging bulimulid genera in South America, with a major concentration of species in Peru and Chile (Breure 1979; Craig 1985). In Chile, *Bostryx* is the most species-rich terrestrial snail genus, and its diversity is strongly associated with coastal localities of the north (Araya 2015; Walther and Gryl 2019). Morphologically, many *Bostryx* species share slender, high-spired shells, but their evolutionary relationships remain incompletely resolved (Araya 2015). Previous multi-locus studies have suggested that *Bostryx* likely represents a complex assemblage requiring continued phylogenetic and taxonomic reassessment, including tests of monophyly and evaluation of deeply divergent lineages (Breure and Romero 2012; Ramirez and Ramírez 2013; Salvador et al. 2023). *Bostryx erythrostoma* (G.B. Sowerby I, 1833) is a Chilean representative of this diverse genus, originally described as *Bulinus erythrostoma* and later treated under *Bostryx* in modern taxonomic syntheses (Breure 2013; Breure and Araujo 2017). Available records indicate that the species occurs along the north–central coast of Chile, including localities in the Atacama Region such as Caldera and Huasco, and extending south to Coquimbo (Stuardo and Vega 1985). Its name usage and synonymy have been compiled in Chilean bulimulid checklists, helping to stabilize nomenclature in regional datasets (Stuardo and Valdovinos 1985).

The Atacama coastal desert also hosts lineages of Bothriembryontidae, particularly the genus *Plectostylus* H. Beck, 1837, a group that has been historically treated in regional taxonomic reviews but that remains underrepresented in molecular datasets (Araya and Catalán 2014). The genus is extant and occurs in Chile and Argentina; species of *Plectostylus* share a minutely rugose, granulate, or striate protoconch, a useful diagnostic character at the genus level (Araya and Catalán 2014). The focal species *Plectostylus broderipii* (G.B. Sowerby I, 1832) is characterized by an elongate-globose, imperforate shell with convex whorls and a pattern of axial and spiral brownish streaks, a large last whorl, and a simple lip (Araya and Catalán 2014). Its reported distribution spans from Iquique to Huasco, and in the Atacama Region it has been found living in sandy substrates near cacti and on rocky outcrops, including localities around Caldera (e.g., El Morro hill and Aguas Verdes) (Araya and Catalán 2014).

Recent progress in high-throughput sequencing has accelerated mitogenome generation across eukaryotes and has highlighted substantial variation in mitochondrial genome architecture and evolutionary dynamics (Zardoya 2020). In molluscs, mitogenomes have proven particularly informative for phylogenetics and comparative evolutionary studies, yet genomic resources remain uneven across major lineages (Ghiselli et al. 2021). For Chilean terrestrial gastropods, and especially for taxonomically complex or geographically restricted groups, complete mitogenomes can provide an essential scaffold to refine phylogenetic placement and enable future population-level applications (Ghiselli et al. 2021).

The objective of this study was to generate additional mitogenome data of two Orthalicoidea land snails from the Atacama Desert of northern Chile, *B. erythrostoma* and *P. broderipii*. Using next-generation sequencing, we assembled and annotated both mitogenomes, characterized their organization and gene content, and evaluated their evolutionary relationships within Helicina using a mitogenome-based phylogenetic framework.

## Material and methods

Specimens of *B. erythrostoma* and *P. broderipii* were collected in the Atacama Region (northern Chile) by Ricardo Catalán-Garrido (Copiapó–Barranquilla, 27°33'20.8"S, 70°51'14.3"W for *B. erythrostoma*; Copiapó–Totoral, 27°47'41.4"S, 71°02'16.7"W for *P. broderipii*), and preserved in 96% ethanol. Identification was performed by Juan Francisco Araya and Ricardo Catalán-Garrido, who identified it based on shell morphology and criteria outlined by Breure (1979) and Beck (1837). Total genomic DNA was extracted from the foot tissue using the Wizard® Genomic DNA Purification Kit (Promega) and the modified Cetyl-trimethylammonium bromide (CTAB) method (Aburto et al. 2020). Paired-end sequencing (150 bp) was performed on an Illumina NovaSeq 6000, generating 63.6 million spots and 19.1 Gb of sequence data for *B. erythrostoma* (SRR37289999), and 39.2 million spots and 11.8 Gb for *P. broderipii* (SRR37291487). Adapter sequences and low-quality bases were removed using Trim Galore (Krueger 2019). Raw reads were inspected with FastQC (Andrews 2010). Mitochondrial assemblies were explored using a combination of *de novo* assemblers, including MEGAHIT (Li et al. 2015) and SPAdes (Bankevich et al. 2012), and a mitochondrion-targeted approach using GetOrganelle (animal_mt settings) (Jin et al. 2020). Candidate mitochondrial contigs were identified by similarity searches using BLAST+ (Camacho et al. 2009). An independent whole-read *de novo* assembly generated with SPAdes was compared with each final mitogenome assembly to assess assembly consistency. A single mitochondrial contig was recovered for each species, and the resulting assemblies were compared across their full lengths to evaluate overall concordance. To validate the final mitogenome assemblies, quality-filtered reads were mapped back to each assembly with BWA-MEM (Li and Durbin 2010). Mapping files were processed with SAMtools (Li et al. 2009) to obtain summary statistics and coverage profiles, including breadth of coverage, depth, and mapping quality. Regions showing local coverage variation were further inspected by visual examination of the read-mapping profiles and comparison with the independent SPAdes assemblies to evaluate local assembly support.

Mitochondrial genomes were initially annotated with MITOS2 (metazoan mode) (Donath et al. 2019) and manually curated by checking gene boundaries and start/stop codons, with special attention to boundary refinement in regions involving rRNA–tRNA adjacency/overlap. As an independent validation, the assemblies were also annotated with MitoFinder (Allio et al. 2020), and the resulting annotations were compared with the manually curated MITOS2 annotations to evaluate gene complement, gene order, strand orientation, and gene-boundary consistency. To facilitate direct comparison between species, both mitogenomes were reoriented to start at cox1; this rotation does not alter gene content or annotation, but standardizes the coordinate system across taxa. Gene-feature summary tables were derived from the final GenBank annotations using custom Python scripts implemented with Biopython (Cock et al. 2009) and pandas to (i) extract CDS, tRNA, and rRNA features, (ii) report strand, feature coordinates, and lengths, (iii) compute start and stop codons for protein-coding genes (PCGs), and (iv) calculate intergenic nucleotides between adjacent features (positive values = spacers; negative values = overlaps; 0 = adjacency). Transfer RNA annotations were further evaluated using ARAGORN (Laslett and Canback 2004) and tRNAscan-SE (Chan et al. 2021), and representative tRNA secondary structures were visualized with VARNA (Darty et al. 2009). Circular genome visualization was performed with Proksee (Grant et al. 2023), and genome statistics (e.g., sequence summaries and base composition) were computed using SeqKit and custom Python scripts. The new mitogenomes have been deposited in the NCBI GenBank database under accession numbers PX617854 (*B. erythrostoma*) and PX977766 (*P. broderipii*). Each of the 13 mitochondrial PCGs was extracted individually and translated into amino-acid sequences. Amino-acid alignments were generated separately for each gene, and codon-aware nucleotide alignments were subsequently produced from the corresponding amino-acid alignments using PAL2NAL (Suyama et al. 2006). Individual codon-aware nucleotide alignments and amino-acid alignments were concatenated into the final nucleotide and amino-acid supermatrices, respectively.

Phylogenetic inference was conducted using a concatenated nucleotide dataset of the 13 PCGs (cox1–3, cob, atp6, atp8, nad1–6, and nad4L), combined into a supermatrix and analyzed under a codon-position partition scheme. Maximum-likelihood (ML) phylogenetic analyses were performed in IQ-TREE v. 2.4.0 (Nguyen et al. 2015). The revised dataset comprised 19 taxa, including two systellommatophoran outgroups *Imerinia grandidieri* (Crosse & P. Fischer, 1871) and *Eleutherocaulis alte* (A. Férussac, 1822), 39 codon-position partitions, and 11,829 aligned nucleotide sites (Suppl. material 1: table S1). Model and partition-scheme selection for the nucleotide dataset were performed in IQ-TREE v. 2.4.0 using ModelFinder under the Bayesian Information Criterion. The initial 39 gene/codon-position partitions were optimized into a 20-partition scheme, which was subsequently used for the final ML analysis. Branch support was estimated using 1000 ultrafast bootstrap (UFBoot) and 1000 SH-aLRT replicates. To further assess the robustness of the inferred relationships, the same set of 13 mitochondrial PCGs was also analyzed as amino-acid sequences. The best-fitting amino acid substitution model (mtZOA+F+I+G4) was selected using ModelFinder Plus (MFP) according to the Bayesian Information Criterion. Branch support was estimated using 1000 ultrafast bootstrap and 1000 SH-aLRT replicates. Bayesian inference was additionally performed in MrBayes v. 3.2.7 (Ronquist et al. 2012) using the same partitioning scheme, with two independent runs of four Markov chains each (three heated and one cold) for 2,000,000 generations, sampling every 1000 generations. Convergence was assessed by examining the average standard deviation of split frequencies (< 0.01), and the first 25% of samples were discarded as burn-in. A majority-rule consensus tree was then generated to estimate posterior probabilities (PP) of nodes.

## Results

**Figure 1.**
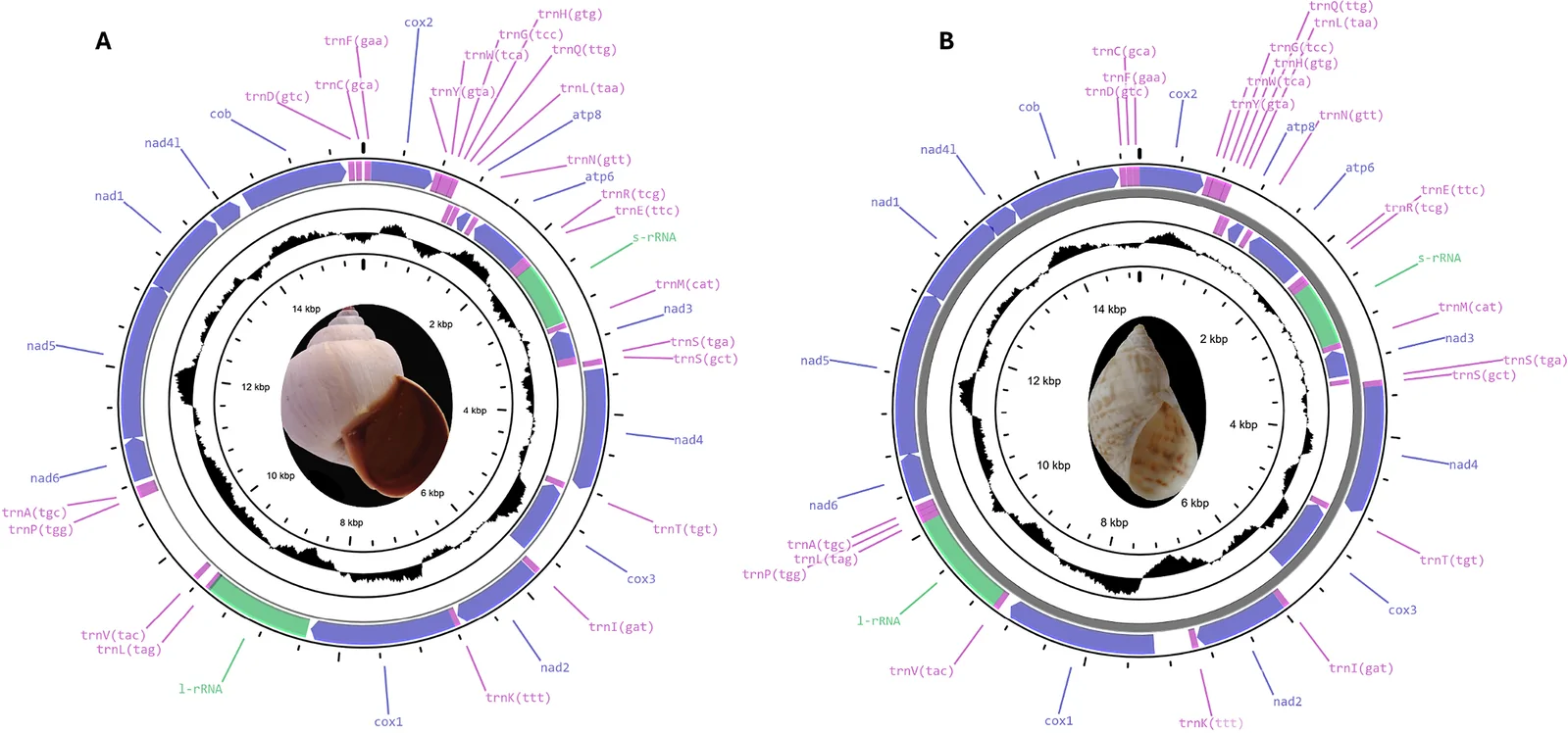
Circular maps of the mitochondrial genomes of two Orthalicoidea land snails from northern Chile. **A**. *Bostryx erythrostoma* (GenBank PX617854), shown in reverse-complemented orientation relative to its deposited GenBank record for direct comparison with **B**. *Plectostylus broderipii* (GenBank PX977766). Protein-coding genes are shown in blue, tRNAs in purple, and rRNAs in green; tRNA labels include anticodons. The two concentric gene tracks indicate genes encoded on opposite strands. The inner histogram shows nucleotide-composition variation across the genome, and the innermost scale indicates genome position (kb). Shell photographs are shown at the center of each map.

We obtained two circular mitochondrial genomes of *Bostryx erythrostoma* (GenBank PX617854) and *Plectostylus broderipii* (GenBank PX977766). For comparative purposes, the *B. erythrostoma* mitogenome was reverse-complemented relative to its GenBank accession (PX617854) and analyzed in the same orientation as that of *P. broderipii*. The mitogenome of *B. erythrostoma* is 15,525 bp long with an overall A+T content of 69.75%, whereas that of *P. broderipii* is 14,978 bp long with 72.08% A+T (Fig. 1; Suppl. material 1: table S3). Both genomes contain the typical metazoan mitochondrial gene complement (37 genes), comprising 13 PCGs, 22 tRNAs, and two rRNAs, plus non-coding intervals of variable length (Suppl. material 1: table S2). The detailed annotation (Suppl. material 1: table S2) also reveals several gene overlaps involving both PCGs and tRNAs. For example, in *P. broderipii*, overlaps occur between nad1–nad4L (23 bp) and nad5–nad6 (8 bp), as well as among adjacent tRNAs such as trnP–trnA (3 bp) and trnW–trnY (6 bp). In *B. erythrostoma*, overlaps are observed between rrnL–trnL1 (26 bp), nad5–nad6 (5 bp), and among tRNAs such as trnH–trnG (4 bp) and trnW–trnY (7 bp) (Suppl. material 1: table S2). Intergenic regions were common in both mitogenomes. In *B. erythrostoma*, we identified 23 intergenic regions totaling 1489 bp (~9.59% of the genome), ranging from 1 to 959 bp; the largest non-coding interval (959 bp) lies between trnV(tac) and trnP(tgg) (Fig. 1; Suppl. material 1: table S5). In *P. broderipii*, 21 intergenic regions were recovered, totaling 949 bp (~6.34% of the genome) and ranging from 1 to 370 bp; the largest interval (370 bp) occurs between trnK(ttt) and cox1, followed by a second large non-coding segment (190 bp) between cox3 and trnI(gat) (Fig. 1; Suppl. material 1: table S5). Gene overlaps were also detected, with nine overlaps in *B. erythrostoma* (maximum 26 bp) and ten overlaps in *P. broderipii* (maximum 23 bp) (Suppl. material 1: table S5). Under the standardized orientation used here, the nucleotide composition of *B. erythrostoma* is A = 5953, T = 4876, C = 2957, G = 1739, corresponding to a negative AT skew (−0.099) and positive GC skew (0.259). Similarly, *P. broderipii* has A = 4853, T = 5943, C = 1946, G = 2236, with a negative AT skew (−0.101) and positive GC skew (0.069) (Suppl. material 1: table S3). Thus, although both mitogenomes are compositionally similar in their high A+T content, they show the same skew direction, with differences in magnitude between species.

PCGs comprise most of each mitogenome, totaling 10,813 bp in *B. erythrostoma* (~69.65% of the genome) and 10,867 bp in *P. broderipii* (~72.55%) (Suppl. material 1: table S4). Accordingly, both species showed the same strand distribution of PCGs and tRNAs, despite contrasting AT and GC skews. Accordingly, nine PCGs are encoded on one strand and four on the opposite strand in both species (Fig. 1; Suppl. material 1: tables S2, S4). Start codons were mostly canonical, but some alternative initiators occurred. In *B. erythrostoma*, PCGs initiated predominantly with ATG (7/13), with additional use of ATT (3/13), ATA (2/13), and TTG (1/13). In *P. broderipii*, ATG was also most frequent (8/13), with ATT (3/13) and TTG (2/13) as alternatives (Suppl. material 1: table S6). Termination codons were mainly TAA and TAG in both species; however, *P. broderipii* includes PCGs with incomplete stop codons (notably nad3, cox3, and nad2), consistent with post-transcriptional completion via polyadenylation (Suppl. material 1: table S7). Excluding start and stop codons, we recovered 3578 sense codons in *B. erythrostoma* and 3598 in *P. broderipii*, reflecting broadly comparable coding capacity despite differences in genome size. Independent annotation with MitoFinder confirmed the manually curated MITOS2 annotation for both mitogenomes. All 13 PCGs, 22 tRNAs, two rRNAs, gene order, and strand orientation were recovered consistently by both approaches. Differences were restricted to minor boundary refinements (1–27 bp), primarily affecting a small number of tRNA genes and one to three PCGs (CYTB in *B. erythrostoma*; ND2, ND3, and COX3 in *P. broderipii*), while preserving identical gene content and overall mitochondrial genome organization (Suppl. material 1: tables S8, S9). Comparisons were performed after standardizing both annotations to the same cox1-based coordinate system to ensure direct correspondence between genomic positions (Suppl. material 1: table S10).

The 22 tRNA genes account for 1445 bp (~9.31%) in *B. erythrostoma* and 1422 bp (~9.49%) in *P. broderipii* (Suppl. material 1: table S4). tRNA lengths range from 59–85 bp in *B. erythrostoma* and 56–69 bp in *P. broderipii* (Suppl. material 1: table S2). In both mitogenomes, 14 tRNAs are encoded on one strand and eight on the other (Fig. 1; Suppl. material 1: tables S2, S4). The complete set of 22 manually curated tRNA secondary structures for each species is provided in Suppl. materials 2, 3. The two rRNA genes contribute 1867 bp (~12.03%) in *B. erythrostoma* and 1803 bp (~12.04%) in *P. broderipii* (Suppl. material 1: table S3). In *B. erythrostoma*, rrnL spans 1122 bp and rrnS 745 bp, whereas in *P. broderipii* rrnL is 1065 bp and rrnS 738 bp (Suppl. material 1: table S2).

**Figure 2.**
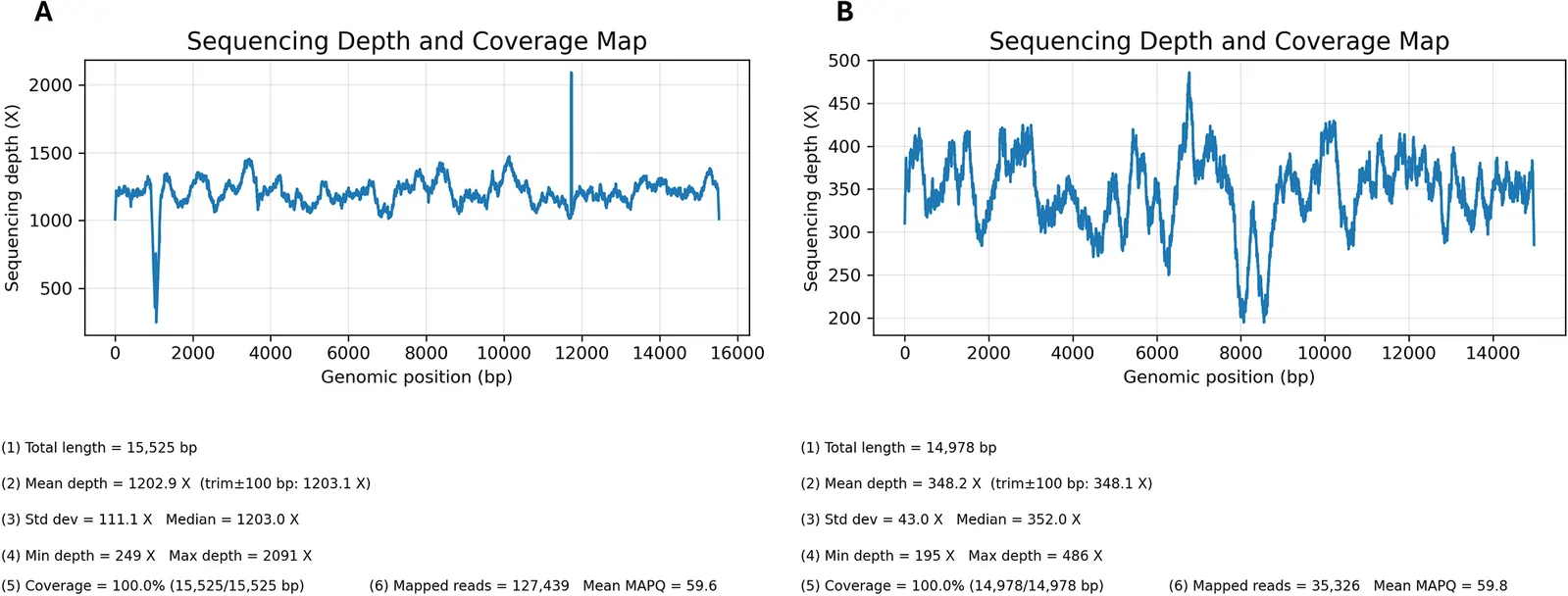
Read-mapping validation of the circular mitochondrial genome assemblies. Sequencing depth profiles along the mitogenome are shown for **A**. *Bostryx erythrostoma*, and **B**. *Plectostylus broderipii*. In both cases, read mapping supports complete breadth of coverage (100%) across the entire circular molecule. Panel summaries report genome length, mean/median depth (×), depth range (min–max), number of mapped reads, and mean mapping quality (MAPQ).

Read mapping against the final assemblies showed complete breadth of coverage (100%) for both mitogenomes, with high mean sequencing depth (mean depth 1202.9× in *B. erythrostoma* and 348.2× in *P. broderipii*) and consistently high mapping quality (mean MAPQ ~59–60) (Fig. 2). Although most of both assemblies was strongly supported, a localized region of the *B. erythrostoma* mitogenome showed reduced coverage and complex read-alignment patterns, as described below. The mapped-read BAM file supporting Fig. 2A and its corresponding index (.bai) are provided as Suppl. materials 6, 7. In *B. erythrostoma*, read-mapping inspection identified a localized region with reduced coverage and clustered soft-clipped alignments. The independent SPAdes assembly produced an alternative reconstruction of the corresponding region, but this reconstruction did not eliminate the local coverage depression or resolve the sequence unequivocally. The remainder of the mitogenome showed consistently high read support and concordance between assembly approaches.

**Figure 3.**
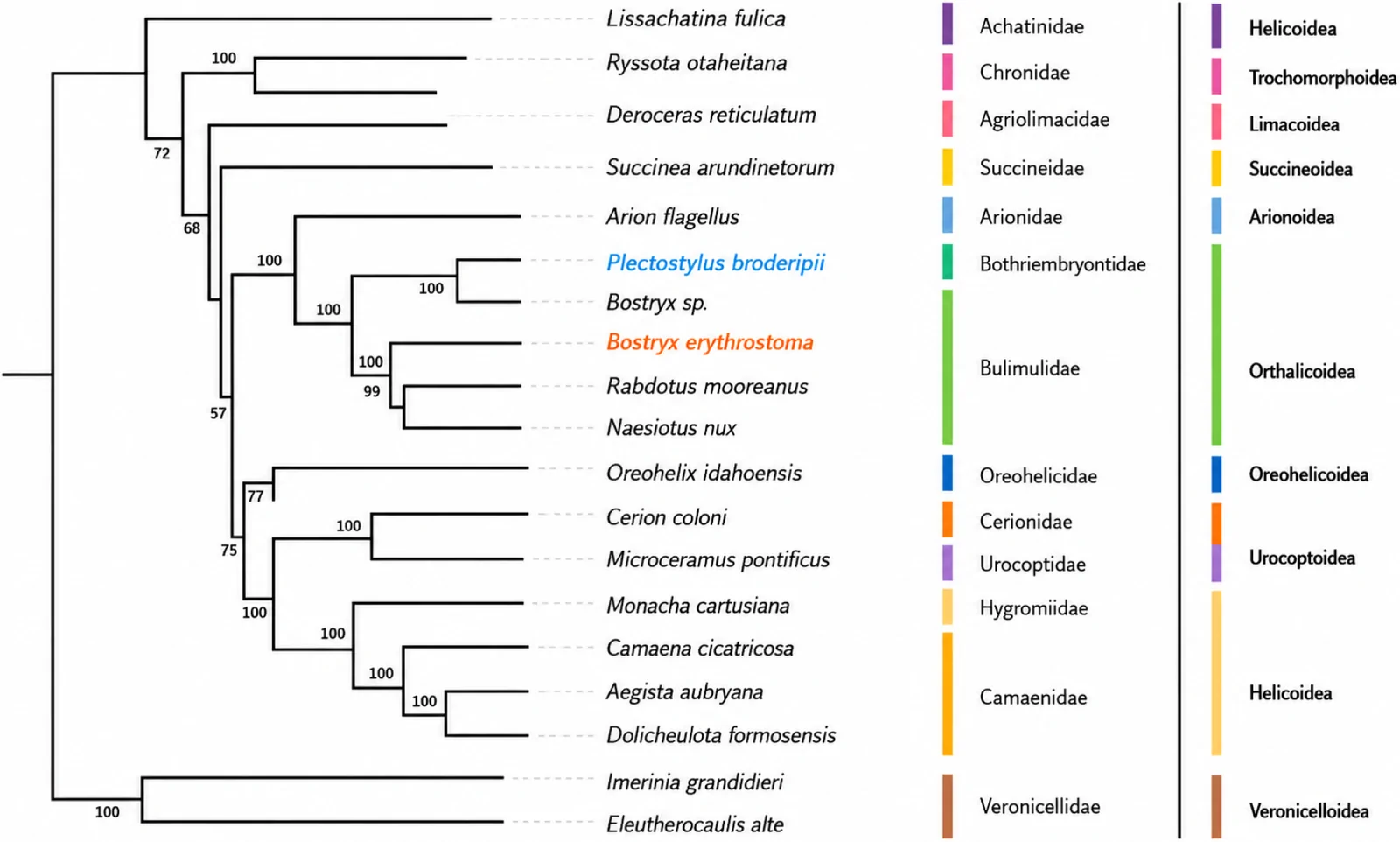
Maximum-likelihood phylogeny inferred from the concatenated, codon-aligned nucleotide supermatrix of 13 mitochondrial protein-coding genes. The analysis includes 19 mitogenomes, with *Imerinia grandidieri* and *Eleutherocaulis alte* used as the outgroups. The tree shows the placement of the two newly generated Orthalicoidea mitogenomes from northern Chile, *P. broderipii* and *B. erythrostoma*, highlighted in blue and orange, respectively. Branch support is given as ultrafast bootstrap values.

**Figure 4.**
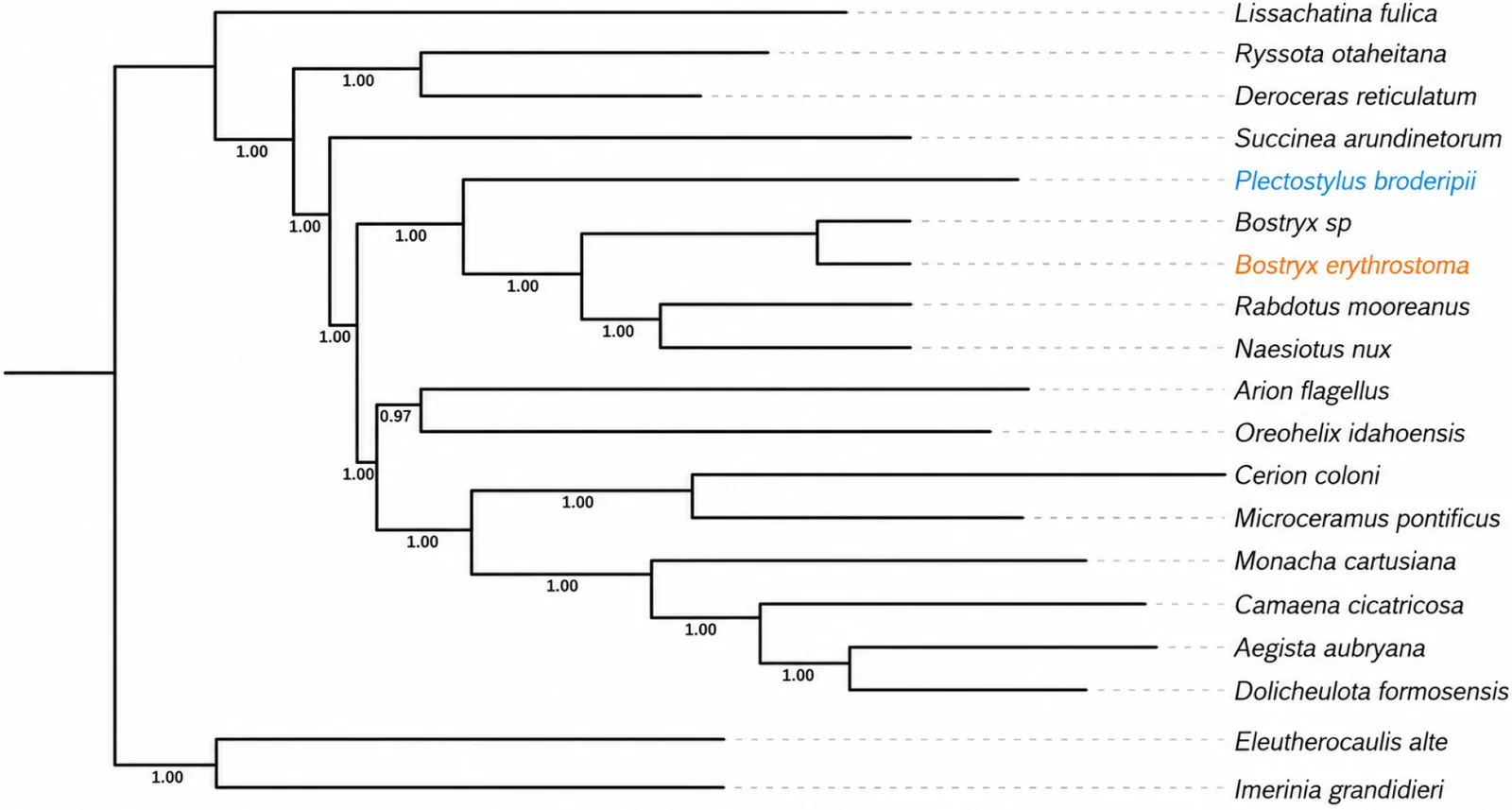
Bayesian phylogeny inferred from the concatenated, codon-aligned nucleotide supermatrix of 13 mitochondrial protein-coding genes. The analysis includes 19 mitogenomes, with *Imerinia grandidieri* and *Eleutherocaulis alte* used as the outgroups. The tree shows the placement of the two newly generated Orthalicoidea mitogenomes from northern Chile, *Plectostylus broderipii* and *Bostryx erythrostoma*, highlighted in blue and orange, respectively. Branch support is shown as Bayesian posterior probabilities on internal branches.

Phylogenetic inference based on the concatenated supermatrix of the 13 mitochondrial PCGs recovered a well-resolved topology with strong support for the focal Orthalicoidea relationships, whereas several deeper nodes showed only moderate support in the ML analysis (Fig. 3). Using *I. grandidieri* and *E. alte* (Systellommatophora) as the outgroups, the remaining taxa (Stylommatophora: Helicina; Suppl. material 1: table S1) formed the ingroup backbone of the tree. Within this framework, the two target sequences, *Bostryx* sp. (PV475493) and *B. erythrostoma* (PX617854), were consistently recovered as sister taxa, forming a clade with maximum support (UFBoot = 100; PP = 1.00) (Figs 3, 4). The clade *Rabdotus mooreanus* (L. Pfeiffer, 1868) and *Naesiotus nux* (Broderip, 1832) (Bulimulidae) was also strongly supported (UFBoot = 99; PP = 1.00), and together with the *Bostryx* sister pair they formed a higher-level clade supported by UFBoot = 100 and PP = 1.00 (Figs 3, 4), reinforcing the stability of the placement of the focal *Bostryx* sequences within the Orthalicoidea taxa sampled (Suppl. material 1: table S1). In addition, *P. broderipii* (Bothriembryontidae) was recovered as the sister lineage to the clade comprising (*Bostryx* sp. + *B. erythrostoma*) and (*Rabdotus mooreanus* + *Naesiotus nux*), with maximum support in both analyses (UFBoot = 100; PP = 1.00) (Figs 3, 4). More generally, the BI-NT tree showed very high PP across the main nodes (most PP = 1.00), with only one deeper node showing slightly lower support (PP = 0.97) (Fig. 4). Finally, the ML amino-acid analysis recovered the same focal Orthalicoidea relationships (Suppl. material 4), supporting the placement of *Plectostylus* and *Bostryx* under an alternative data encoding. Overall, the combination of strongly supported sister-group relationships and the consistent recovery of the focal Orthalicoidea relationships provides a clear and robust phylogenetic context for the target sequences, with key nodes supported by UFBoot values close to or equal to 100 and PP close to or equal to 1.00.

## Discussion and conclusion

The mitochondrial genome assemblies of *Bostryx erythrostoma* (15,525 bp) and *Plectostylus broderipii* (14,978 bp) represent the first mitogenomic resources for these species and provide reference sequences for future comparative analyses of Orthalicoidea land snails. These data also contribute to broader phylogenetic studies of stylommatophoran lineages, for which mitochondrial genomes have proven informative (Gaitán-Espitia et al. 2013; Lin et al. 2016). A localized region of the assembled *B. erythrostoma* mitochondrial genome showed reduced read support during post-assembly read-mapping validation. This region exhibited decreased coverage and an increased number of soft-clipped alignments, and independent assembly strategies did not unequivocally resolve its sequence using short Illumina reads. Consequently, we conservatively consider the mitochondrial genome of *B. erythrostoma* to be nearly complete.

Both *B. erythrostoma* and *P. broderipii* fall within the range of mitogenome sizes typically observed in Orthalicoidea and retain the standard metazoan mitochondrial gene complement (37 genes: 13 PCGs, 22 tRNAs, and 2 rRNAs), indicating broad conservation of overall genomic architecture. The difference in total genome length between the two species appears to be caused by non-coding DNA rather than by major changes in coding-gene content. In *B. erythrostoma*, intergenic DNA is comparatively expanded, with 23 regions totaling 1489 bp (~9.59% of the genome), including a prominent 959-bp interval between trnV(tac) and trnP(tgg). By contrast, *P. broderipii* is more compact, with 21 intergenic regions totaling 949 bp (~6.34%), and its largest spacer (370 bp) occurs between trnK(ttt) and cox1. This pattern is consistent with comparisons to other orthalicoid mitogenomes, including *N. nux* (Hunter et al. 2016) and *R. mooreanus*; it also supports the view that differences in mitogenome size within the superfamily are shaped chiefly by lineage-specific expansion and contraction of intergenic tracts, even when overall gene content remains conserved (Gonzalez et al. 2016). The sampled Orthalicoidea mitogenomes, including *B. erythrostoma*, *P. broderipii*, *Bostryx* sp., *N. nux*, and *R. mooreanus*, retain the same order of the 13 PCGs, reflecting a highly conserved organization consistent with the ancestral/conservative pattern reported by Wang et al. (2025); differences among them are mainly restricted to local tRNA/rRNA rearrangements.

The alternative cox1–rrnL–trnL1–trnV organization observed in *Bostryx erythrostoma* is unlikely to represent an annotation artifact. Independent annotation with MitoFinder reproduced the same gene order obtained after manual curation of the MITOS2 annotation, while ARAGORN and tRNAscan-SE consistently recovered the same tRNA complement and strand orientation. Moreover, the identical organization was independently observed in the published mitogenome of *Bostryx* sp., indicating that this arrangement most likely represents a lineage-specific mitochondrial gene-order feature within the genus rather than an isolated assembly or annotation error. This interpretation is consistent with the remarkable evolutionary plasticity of molluscan mitochondrial genomes, in which gene-order rearrangements—including those involving tRNA genes—are widespread and constitute a major source of structural variation. Within Stylommatophora, such rearrangements have repeatedly been shown to characterize particular evolutionary lineages and may provide additional phylogenetic information when interpreted together with sequence-based evidence (Groenenberg et al. 2017; Xie et al. 2019; Ghiselli et al. 2021). The recurrence of this organization in two independently assembled *Bostryx* mitogenomes further suggests that it may represent a conserved mitochondrial feature of Atacama Desert *Bostryx* lineages, although additional congeneric mitogenomes will be required to determine its broader evolutionary distribution.

The two new mitogenomes are strongly A+T biased (69.75% in *B. erythrostoma*; 72.08% in *P. broderipii*), consistent with the A+T enrichment commonly observed in stylommatophoran mitogenomes, including those examined by Wang et al. (2025). Under the standardized orientation used here, both species also show negative AT skew and positive GC skew, indicating a broadly conserved pattern of compositional asymmetry across Orthalicoidea. A similarly high A+T content is observed in *N. nux* (Hunter et al. 2016).

From an annotation perspective, both mitogenomes exhibit typical metazoan features, with ATG as the predominant initiation codon but recurrent use of alternative start codons (ATT, ATA, and TTG), as also reported in other stylommatophoran mitogenomes (Wang et al. 2025). Incomplete stop codons were detected in *P. broderipii* (nad3, cox3, and nad2), whereas no incomplete stop codons were identified in *B. erythrostoma*, consistent with post-transcriptional completion via polyadenylation. A comparable pattern is also present in *N. nux*, which shows alternative start codons and truncated stop codons in nad3 and nad2 (Hunter et al. 2016). As in other metazoan mitogenomes, however, the precise delimitation of gene boundaries, particularly those adjacent to tRNA genes, remains partly tentative because current annotation algorithms cannot fully account for post-transcriptional RNA processing. Consequently, automated annotations require manual curation and comparative evaluation across related taxa. Accordingly, the gene boundaries reported here, refined through the comprehensive manual curation performed in the present study, should be regarded as the best-supported estimates based on the currently available comparative evidence.

The manually curated secondary structures further indicate that the vast majority of mitochondrial tRNAs exhibit comparable folding patterns in both species, despite minor lineage-specific differences in stem length and loop architecture. Most tRNAs conform to the canonical metazoan cloverleaf structure after manual refinement, whereas only a few genes, particularly trnS2, retain reduced structural elements (Watanabe et al. 2014). Such simplified serine tRNAs have been repeatedly documented across metazoan mitochondrial genomes and are generally regarded as authentic evolutionary features rather than annotation artifacts (Boore et al. 2005). These observations further support the reliability of the annotation strategy adopted in this study, which combined covariance-model prediction with comparative manual curation.

Phylogenetically, the 13-PCG matrix yielded a well-resolved placement of the new Atacama Desert mitogenomes. *Rabdotus mooreanus* and *N. nux*, representing the subfamily Bulimulinae, formed a sister pair, which was recovered as sister to the clade (*Bostryx* sp. + *B. erythrostoma*), representing the subfamily Bostrycinae, whereas *P. broderipii* was recovered as sister to the remaining sampled Orthalicoidea. This placement is informative in the context of the complex taxonomy reported for *Bostryx* (Ramirez and Ramírez 2013) and highlights the need for denser molecular sampling to refine orthalicoid relationships (Salvador et al. 2023). In this context, these first mitogenomes for *B. erythrostoma* and *P. broderipii* provide useful reference points for future integrative phylogenetic work in Bulimulidae and Bothriembryontidae, particularly as complete mitogenomes continue to improve comparative inference in Stylommatophora (Wang et al. 2025). Although some deeper relationships among stylommatophoran superfamilies received lower support or differed slightly between inference methods, these deeper nodes should be interpreted with caution because mitochondrial genomes sampled across distantly related stylommatophoran lineages are expected to exhibit heterogeneous evolutionary rates and compositional heterogeneity, both of which may influence phylogenetic inference at deeper evolutionary scales. Nevertheless, the relationships within Orthalicoidea were consistently recovered in the nucleotide-, amino acid-, and Bayesian-based analyses, supporting the phylogenetic placement of the two newly sequenced taxa.

These mitochondrial genome resources, the complete mitogenome of *Plectostylus broderipii* and the nearly complete mitogenome of *Bostryx erythrostoma*, provide the first reference sequences for these species and will facilitate future comparative and phylogenetic studies of Orthalicoidea land snails, particularly once denser congeneric and taxon sampling becomes available.

## Additional information

### Artificial Intelligence (AI) use

The authors accept full responsibility for the content of the manuscript, including the disclosure of any use of AI.

No AI tools were used in the preparation of this manuscript.

### Author ORCIDs

R. Contreras-Díaz https://orcid.org/0000-0002-5607-811X

R.M. Barahona-Segovia https://orcid.org/0000-0002-1509-2935

J.F. Araya https://orcid.org/0000-0002-4087-9641

M. Arias-Aburto https://orcid.org/0000-0001-5401-2777

### Data availability

The mitochondrial genome sequences are available in GenBank (https://www.ncbi.nlm.nih.gov/genbank/) under the accession numbers PX617854 (*Bostryx erythrostoma*) and PX977766 (*Plectostylus broderipii*). The associated BioProject, BioSample and SRA numbers are PRJNA1425805, SAMN55631320, and SRR37289999 for *B. erythrostoma*, and PRJNA1425958, SAMN55687149, and SRR37291487 for *P. broderipii*, respectively.

## Supplementary materials

Supplementary material 1 Supplementary tables S1–S10 Data type docx Explanation note **table S1**. Taxa and GenBank accession numbers used for phylogenetic inference, including higher-level taxonomy (order, superfamily, and family). **table S2**. Gene annotation features of the mitochondrial genomes of *Plectostylus broderipii* and *Bostryx erythrostoma*, reoriented to start at cox1. **table S3**. Genome size and nucleotide composition. **table S4**. Gene content summary (total length and percent of genome). **table S5**. Intergenic regions and overlaps. **table S6**. Start codons across the 13 PCGs. **table S7**. PCGs annotated with incomplete stop codons (completed by polyadenylation). **table S8**. Comparison of mitochondrial gene annotations obtained by manual curation (MITOS2-based) and MitoFinder for the complete mitogenome of *Plectostylus broderipii*. **table S9**. Comparison of mitochondrial gene annotations obtained by manual curation (MITOS2-based) and MitoFinder for the nearly complete mitochondrial genome *Bostryx erythrostoma*. **table S10**. Comparison of the mitochondrial gene order in the COX1–ND6 region among representative Stylommatophora mitogenomes. Gene orders were oriented according to the transcriptional direction of COX1. https://binary.pensoft.net/file/1761632This dataset is made available under the Open Database License (http://opendatacommons.org/licenses/odbl/1.0/). The Open Database License (ODbL) is a license agreement intended to allow users to freely share, modify, and use this Dataset while maintaining this same freedom for others, provided that the original source and author(s) are credited. Roberto Contreras-Díaz, Rodrigo M. Barahona-Segovia, Ricardo Catalán-Garrido, Juan Francisco Araya, Mariana Arias-Aburto, César Pizarro-Gacitúa

Supplementary material 2 *Bostryx erythrostoma* – curated cloverleaf tRNA structures v5 Data type png Explanation note **figure S1**. Representative mitochondrial tRNA secondary structures of *Bostryx erythrostoma*. Structures were predicted using tRNAscan-SE and visualized with VARNA. Panels show selected tRNA examples for each mitogenome. https://binary.pensoft.net/file/1761633This dataset is made available under the Open Database License (http://opendatacommons.org/licenses/odbl/1.0/). The Open Database License (ODbL) is a license agreement intended to allow users to freely share, modify, and use this Dataset while maintaining this same freedom for others, provided that the original source and author(s) are credited. Roberto Contreras-Díaz, Rodrigo M. Barahona-Segovia, Ricardo Catalán-Garrido, Juan Francisco Araya, Mariana Arias-Aburto, César Pizarro-Gacitúa

Supplementary material 3 *Plectostylus broderipii* – curated cloverleaf tRNA structures v5 Data type png Explanation note **figure S2**. Representative mitochondrial tRNA secondary structures of *Plectostylus broderipii*. Structures were predicted using tRNAscan-SE and visualized with VARNA. Panels show selected tRNA examples for each mitogenome. https://binary.pensoft.net/file/1761634This dataset is made available under the Open Database License (http://opendatacommons.org/licenses/odbl/1.0/). The Open Database License (ODbL) is a license agreement intended to allow users to freely share, modify, and use this Dataset while maintaining this same freedom for others, provided that the original source and author(s) are credited. Roberto Contreras-Díaz, Rodrigo M. Barahona-Segovia, Ricardo Catalán-Garrido, Juan Francisco Araya, Mariana Arias-Aburto, César Pizarro-Gacitúa

Supplementary material 4 Maximum-likelihood phylogeny inferred from the concatenated amino-acid supermatrix of the 13 mitochondrial protein-coding genes Data type jpg Explanation note **figure S3**. Maximum-likelihood phylogeny inferred from the concatenated amino-acid supermatrix of the 13 mitochondrial protein-coding genes. The analysis includes 19 mitogenomes, with *Imerinia grandidieri* and *Eleutherocaulis alte* used as the outgroups. The tree shows the placement of the two newly generated Orthalicoidea mitogenomes from northern Chile, *Plectostylus broderipii* (blue) and *Bostryx erythrostoma* (orange). Branch support is shown as ultrafast bootstrap (UFBoot) values on nodes. https://binary.pensoft.net/file/1761635This dataset is made available under the Open Database License (http://opendatacommons.org/licenses/odbl/1.0/). The Open Database License (ODbL) is a license agreement intended to allow users to freely share, modify, and use this Dataset while maintaining this same freedom for others, provided that the original source and author(s) are credited. Roberto Contreras-Díaz, Rodrigo M. Barahona-Segovia, Ricardo Catalán-Garrido, Juan Francisco Araya, Mariana Arias-Aburto, César Pizarro-Gacitúa

Supplementary material 5 Manual curation of tRNA secondary structures Data type docx https://binary.pensoft.net/file/1761636This dataset is made available under the Open Database License (http://opendatacommons.org/licenses/odbl/1.0/). The Open Database License (ODbL) is a license agreement intended to allow users to freely share, modify, and use this Dataset while maintaining this same freedom for others, provided that the original source and author(s) are credited. Roberto Contreras-Díaz, Rodrigo M. Barahona-Segovia, Ricardo Catalán-Garrido, Juan Francisco Araya, Mariana Arias-Aburto, César Pizarro-Gacitúa

Supplementary material 6 Data S1 Data type bam Explanation note Coordinate-sorted BAM file containing the reads mapped to the final *Bostryx erythrostoma* mitochondrial genome assembly. https://binary.pensoft.net/file/1761637This dataset is made available under the Open Database License (http://opendatacommons.org/licenses/odbl/1.0/). The Open Database License (ODbL) is a license agreement intended to allow users to freely share, modify, and use this Dataset while maintaining this same freedom for others, provided that the original source and author(s) are credited. Roberto Contreras-Díaz, Rodrigo M. Barahona-Segovia, Ricardo Catalán-Garrido, Juan Francisco Araya, Mariana Arias-Aburto, César Pizarro-Gacitúa

Supplementary material 7 Data S2 Data type bai Explanation note Corresponding index (.bai) to Data S1. https://binary.pensoft.net/file/1761638This dataset is made available under the Open Database License (http://opendatacommons.org/licenses/odbl/1.0/). The Open Database License (ODbL) is a license agreement intended to allow users to freely share, modify, and use this Dataset while maintaining this same freedom for others, provided that the original source and author(s) are credited. Roberto Contreras-Díaz, Rodrigo M. Barahona-Segovia, Ricardo Catalán-Garrido, Juan Francisco Araya, Mariana Arias-Aburto, César Pizarro-Gacitúa
