# Supplementary material for: Mitochondrial genomes and phylogenomic analysis of Plectostylus broderipii and Bostryx erythrostoma (Gastropoda, Stylommatophora, Orthalicoidea) from the Atacama Desert, Chile
Source: Zookeys. 2026 Sep 1;1291:1–18. doi: 10.3897/zookeys.1291.197983 (PMC13555134; doi:10.3897/zookeys.1291.197983)
Supplement: Supplementary file 1 [file zookeys-1291-001_article-197983__-s001.docx]

**Table S1.** Taxa and GenBank accession numbers used for phylogenetic inference, including higher-level taxonomy (order, superfamily, and family).

| **Taxón** | **Accession** | **Order** | **Suborder** | **Superfamily** | **Family** |
| --- | --- | --- | --- | --- | --- |
| *Imerinia grandidieri* | JN619347 | Systellommatophora | — | Veronicelloidea | Veronicellidae |
| *Eleutherocaulis alte* | PP716841 | Systellommatophora | — | Veronicelloidea | Veronicellidae |
| *Ryssota otaheitana* | NC_044784 | Stylommatophora | Helicina | Trochomorphoidea | Chronidae |
| *Deroceras reticulatum* | NC_035495 | Stylommatophora | Helicina | Limacoidea | Agriolimacidae |
| *Arion flagellus* | NC_073101 | Stylommatophora | Helicina | Arionoidea | Arionidae |
| *Lissachatina fulica (= Achatina fulica)* | NC_024601 | Stylommatophora | Helicina | Achatinoidea | Achatinidae |
| *Plectostylus broderipii* | **PX977766** | Stylommatophora | Helicina | Orthalicoidea | Bothriembryontidae |
| *Bostryx sp.* | PV475493 | Stylommatophora | Helicina | Orthalicoidea | Bulimulidae |
| *Bostryx erythrostoma* | **PX617854** | Stylommatophora | Helicina | Orthalicoidea | Bulimulidae |
| *Rabdotus mooreanus* | NC_082226 | Stylommatophora | Helicina | Orthalicoidea | Bulimulidae |
| *Naesiotus nux* | NC_028553 | Stylommatophora | Helicina | Orthalicoidea | Bulimulidae |
| *Succinea arundinetorum* | NC_068814 | Stylommatophora | Helicina | Succineoidea | Succineidae |
| *Oreohelix idahoensis* | NC_043790 | Stylommatophora | Helicina | Oreohelicoidea | Oreohelicidae |
| *Cerion coloni* | NC_063566 | Stylommatophora | Helicina | Urocoptoidea | Cerionidae |
| *Microceramus pontificus* | NC_036381 | Stylommatophora | Helicina | Urocoptoidea | Urocoptidae |
| *Monacha cartusiana* | NC_072058 | Stylommatophora | Helicina | Helicoidea | Hygromiidae |
| *Camaena cicatricosa* | NC_025511 | Stylommatophora | Helicina | Helicoidea | Camaenidae |
| *Aegista aubryana* | NC_029419 | Stylommatophora | Helicina | Helicoidea | Camaenidae |
| *Dolicheulota formosensis* | NC_027493 | Stylommatophora | Helicina | Helicoidea | Camaenidae |

**Table S2.** Gene annotation features of the mitochondrial genomes of *Plectostylus broderipii* and *Bostryx erythrostoma*, reoriented to start at cox1.

Note: Intergenic bp from previous feature indicates the number of nucleotides between adjacent features; positive values indicate spacers, negative values indicate overlaps, and 0 indicates adjacent features. Start and stop codons are shown only for protein-coding genes. For comparative purposes, both mitochondrial genomes are presented starting from the COX1 gene and following its transcriptional orientation. In the original GenBank accession, the mitochondrial genome of *Bostryx erythrostoma* is presented in the opposite orientation. This standardized presentation does not affect gene content or annotation but facilitates direct comparison of gene order, gene orientation, start and stop codons, and intergenic regions between the two species. Leucine and serine tRNA genes are designated as trnL1/trnL2 and trnS1/trnS2, respectively, following standard mitochondrial nomenclature.

***Plectostylus broderipii***

| **Feature** | **Gene** | **Product** | **Start** | **End** | **Strand** | **Length (bp)** | **Start codon** | **Stop codon** | **Previous feature** | **Intergenic bp from previous** |
| --- | --- | --- | --- | --- | --- | --- | --- | --- | --- | --- |
| CDS | cox1 | cytochrome c oxidase subunit 1 | 1 | 1536 | + | 1536 | TTG | TAA |  |  |
| tRNA | trnV(tac) | tRNA-Val | 1618 | 1681 | + | 64 |  |  | cox1 | 81 |
| rRNA | l-rRNA (rrnL) | large ribosomal RNA | 1680 | 2744 | + | 1065 |  |  | trnV(tac) | -2 |
| tRNA | trnL1(tag) | tRNA-Leu | 2745 | 2812 | + | 68 |  |  | l-rRNA | 0 |
| tRNA | trnP(tgg) | tRNA-Pro | 2810 | 2872 | + | 63 |  |  | trnL1(tag) | -3 |
| tRNA | trnA(tgc) | tRNA-Ala | 2870 | 2933 | + | 64 |  |  | trnP(tgg) | -3 |
| CDS | nad6 | NADH dehydrogenase subunit 6 | 2980 | 3453 | + | 474 | ATT | TAA | trnA(tgc) | 46 |
| CDS | nad5 | NADH dehydrogenase subunit 5 | 3446 | 5113 | + | 1668 | ATG | TAA | nad6 | -8 |
| CDS | nad1 | NADH dehydrogenase subunit 1 | 5106 | 6029 | + | 924 | ATG | TAG | nad5 | -8 |
| CDS | nad4l | NADH dehydrogenase subunit 4L | 6007 | 6306 | + | 300 | ATG | TAA | nad1 | -23 |
| CDS | cob | cytochrome b | 6308 | 7426 | + | 1119 | TTG | TAA | nad4l | 1 |
| tRNA | trnD(gtc) | tRNA-Asp | 7436 | 7500 | + | 65 |  |  | cob | 9 |
| tRNA | trnC(gca) | tRNA-Cys | 7504 | 7568 | + | 65 |  |  | trnD(gtc) | 3 |
| tRNA | trnF(gaa) | tRNA-Phe | 7570 | 7635 | + | 66 |  |  | trnC(gca) | 1 |
| CDS | cox2 | cytochrome c oxidase subunit 2 | 7637 | 8296 | + | 660 | ATG | TAA | trnF(gaa) | 1 |
| tRNA | trnY(gta) | tRNA-Tyr | 8303 | 8371 | + | 69 |  |  | cox2 | 6 |
| tRNA | trnW(tca) | tRNA-Trp | 8366 | 8433 | + | 68 |  |  | trnY(gta) | -6 |
| tRNA | trnG(tcc) | tRNA-Gly | 8434 | 8499 | + | 66 |  |  | trnW(tca) | 0 |
| tRNA | trnH(gtg) | tRNA-His | 8495 | 8561 | + | 67 |  |  | trnG(tcc) | -5 |
| tRNA | trnQ(ttg) | tRNA-Gln | 8566 | 8621 | - | 56 |  |  | trnH(gtg) | 4 |
| tRNA | trnL2(taa) | tRNA-Leu | 8624 | 8686 | - | 63 |  |  | trnQ(ttg) | 2 |
| CDS | atp8 | ATP synthase F0 subunit 8 | 8716 | 8871 | - | 156 | ATT | TAA | trnL2(taa) | 29 |
| tRNA | trnN(gtt) | tRNA-Asn | 8923 | 8990 | - | 68 |  |  | atp8 | 51 |
| CDS | atp6 | ATP synthase F0 subunit 6 | 9013 | 9666 | - | 654 | ATG | TAA | trnN(gtt) | 22 |
| tRNA | trnR(tcg) | tRNA-Arg | 9732 | 9795 | - | 64 |  |  | atp6 | 65 |
| tRNA | trnE(ttc) | tRNA-Glu | 9793 | 9855 | - | 63 |  |  | trnR(tcg) | -3 |
| rRNA | s-rRNA | small ribosomal RNA | 9856 | 10593 | - | 738 |  |  | trnE(ttc) | 0 |
| tRNA | trnM(cat) | tRNA-Met | 10596 | 10662 | - | 67 |  |  | s-rRNA | 2 |
| CDS | nad3 | NADH dehydrogenase subunit 3 | 10663 | 10981 | - | 319 | ATT | T-- | trnM(cat) | 0 |
| tRNA | trnS2(tga) | tRNA-Ser | 11022 | 11077 | - | 56 |  |  | nad3 | 40 |
| tRNA | trnS1(gct) | tRNA-Ser | 11076 | 11134 | + | 59 |  |  | trnS2(tga) | -2 |
| CDS | nad4 | NADH dehydrogenase subunit 4 | 11135 | 12448 | + | 1314 | ATG | TAA | trnS1(gct) | 0 |
| tRNA | trnT(tgt) | tRNA-Thr | 12472 | 12539 | - | 68 |  |  | nad4 | 23 |
| CDS | cox3 | cytochrome c oxidase subunit 3 | 12540 | 13362 | - | 823 | ATG | T-- | trnT(tgt) | 0 |
| tRNA | trnI(gat) | tRNA-Ile | 13553 | 13618 | + | 66 |  |  | cox3 | 190 |
| CDS | nad2 | NADH dehydrogenase subunit 2 | 13620 | 14539 | + | 920 | ATG | TA- | trnI(gat) | 1 |
| tRNA | trnK(ttt) | tRNA-Lys | 14542 | 14608 | + | 67 |  |  | nad2 | 2 |

***Bostryx erythrostoma***

| **Feature** | **Gene** | **Product** | **Start** | **End** | **Strand** | **Length (bp)** | **Start codon** | **Stop codon** | **Previous feature** | **Intergenic bp from previous** |
| --- | --- | --- | --- | --- | --- | --- | --- | --- | --- | --- |
| CDS | cox1 | cytochrome c oxidase subunit 1 | 1 | 1527 | − | 1527 | TTG | TAA |  | — |
| rRNA | l-rRNA (rrnL) | **large ribosomal RNA** | 14374 | 15495 | − | 1122 |  |  | cox1 | 30 |
| tRNA | trnL1(tag) | tRNA-Leu | 14334 | 14399 | − | 66 |  |  | l-rRNA | −26 |
| tRNA | trnV(tac) | tRNA-Val | 14169 | 14230 | − | 62 |  |  | trnL1(tag) | 103 |
| tRNA | trnP(tgg) | tRNA-Pro | 13147 | 13209 | − | 63 |  |  | trnV(tac) | 959 |
| tRNA | trnA(tgc) | tRNA-Ala | 13081 | 13145 | − | 65 |  |  | trnP(tgg) | 1 |
| CDS | nad6 | NADH dehydrogenase subunit 6 | 12578 | 13033 | − | 456 | ATT | TAA | trnA(tgc) | 47 |
| CDS | nad5 | NADH dehydrogenase subunit 5 | 10915 | 12582 | − | 1668 | ATT | TAA | nad6 | −5 |
| CDS | nad1 | NADH dehydrogenase subunit 1 | 10028 | 10933 | − | 906 | ATG | TAG | nad5 | −19 |
| CDS | nad4l | NADH dehydrogenase subunit 4L | 9712 | 10017 | − | 306 | ATG | TAA | nad1 | 10 |
| CDS | cob | cytochrome b | 8507 | 9626 | − | 1120 | ATG | TAA | nad4l | 85 |
| tRNA | trnD(gtc) | tRNA-Asp | 8422 | 8485 | − | 64 |  |  | cob | 21 |
| tRNA | trnC(gca) | tRNA-Cys | 8345 | 8404 | − | 60 |  |  | trnD(gtc) | 17 |
| tRNA | trnF(gaa) | tRNA-Phe | 8250 | 8315 | − | 66 |  |  | trnC(gca) | 29 |
| CDS | cox2 | cytochrome c oxidase subunit 2 | 7581 | 8246 | − | 666 | ATG | TAA | trnF(gaa) | 3 |
| tRNA | trnY(gta) | tRNA-Tyr | 7510 | 7580 | − | 71 |  |  | cox2 | 0 |
| tRNA | trnW(tca) | tRNA-Trp | 7453 | 7516 | − | 64 |  |  | trnY(gta) | −7 |
| tRNA | trnG(tcc) | tRNA-Gly | 7387 | 7452 | − | 66 |  |  | trnW(tca) | 0 |
| tRNA | trnH(gtg) | tRNA-His | 7329 | 7390 | − | 62 |  |  | trnG(tcc) | −4 |
| tRNA | trnQ(ttg) | tRNA-Gln | 7267 | 7330 | + | 64 |  |  | trnH(gtg) | −2 |
| tRNA | trnL2(taa) | tRNA-Leu | 7187 | 7255 | + | 69 |  |  | trnQ(ttg) | 11 |
| CDS | atp8 | ATP synthase F0 subunit 8 | 7043 | 7174 | + | 132 | ATT | TAA | trnL2(taa) | 12 |
| tRNA | trnN(gtt) | tRNA-Asn | 6943 | 7008 | + | 66 |  |  | atp8 | 34 |
| CDS | atp6 | ATP synthase F0 subunit 6 | 6273 | 6926 | + | 654 | ATG | TAA | trnN(gtt) | 16 |
| tRNA | trnR(tcg) | tRNA-Arg | 6207 | 6270 | + | 64 |  |  | atp6 | 2 |
| tRNA | trnE(ttc) | tRNA-Glu | 6140 | 6205 | + | 66 |  |  | trnR(tcg) | 1 |
| rRNA | s-rRNA | **small ribosomal RNA** | 5401 | 6145 | + | 745 |  |  | trnE(ttc) | −6 |
| tRNA | trnM(cat) | tRNA-Met | 5327 | 5389 | + | 63 |  |  | s-rRNA | 11 |
| CDS | nad3 | NADH dehydrogenase subunit 3 | 4982 | 5326 | + | 345 | ATA | TAA | trnM(cat) | 0 |
| tRNA | trnS2(tga) | tRNA-Ser | 4897 | 4981 | + | 85 |  |  | nad3 | 0 |
| tRNA | trnS1(gct) | tRNA-Ser | 4847 | 4907 | − | 61 |  |  | trnS2(tga) | −11 |
| CDS | nad4 | NADH dehydrogenase subunit 4 | 3510 | 4805 | − | 1296 | ATA | TAG | trnS1(gct) | 41 |
| tRNA | trnT(tgt) | tRNA-Thr | 3448 | 3518 | + | 71 |  |  | nad4 | −9 |
| CDS | cox3 | cytochrome c oxidase subunit 3 | 2628 | 3446 | + | 819 | ATG | TAA | trnT(tgt) | 1 |
| tRNA | trnI(gat) | tRNA-Ile | 2518 | 2585 | − | 68 |  |  | cox3 | 42 |
| CDS | nad2 | NADH dehydrogenase subunit 2 | 1593 | 2510 | − | 918 | ATG | TAA | trnI(gat) | 7 |
| tRNA | trnK(ttt) | tRNA-Lys | 1528 | 1586 | − | 59 |  |  | nad2 | 6 |

**Note**: For comparative purposes, both mitochondrial genomes are presented starting from the COX1 gene and following its transcriptional orientation. This standardized presentation facilitates direct comparison of gene order, gene orientation, coding features, and intergenic regions between the two species. Leucine and serine tRNA genes are designated as trnL1/trnL2 and trnS1/trnS2, respectively, following standard mitochondrial nomenclature.

**Table S3**. Genome size and nucleotide composition

| **Metric** | ***Bostryx erythrostoma* (PX617854; reverse-complemented for analysis)** | ***Plectostylus broderipii* (PX977766)** |
| --- | --- | --- |
| Genome length (bp) | 15,525 | 14,978 |
| A | 5,953 | 4,853 |
| T | 4,876 | 5,943 |
| C | 2,957 | 1,946 |
| G | 1,739 | 2,236 |
| A+T (%) | 69.75 | 72.08 |
| AT skew | -0.099 | −0.101 |
| GC skew | 0.259 | 0.069 |

**Table S4**. Gene content summary (total length and percent of genome)

| **Feature set** | ***Bostryx***  **total (bp)** | ***Bostryx***  **(% genome)** | ***Plectostylus* total (bp)** | ***Plectostylus* (% genome)** |
| --- | --- | --- | --- | --- |
| Protein-coding genes (PCGs) | 10,813 | 69.65 | 10,867 | 72.55 |
| tRNAs | 1,445 | 9.31 | 1,422 | 9.49 |
| rRNAs | 1,867 | 12.03 | 1,803 | 12.04 |

**Table S5**. Intergenic regions and overlaps

| **Metric** | ***Bostryx*** | ***Plectostylus*** |
| --- | --- | --- |
| Intergenic regions (n) | 23 | 21 |
| Total intergenic length (bp) | 1,489 | 949 |
| Largest intergenic region (bp) | 959 | 370 |
| Largest intergenic (between genes) | trnV(tac)-trnP(tgg) | trnK(ttt)–cox1 |
| Overlapping regions (n) | 9 | 10 |
| Largest overlap (bp) | 26 | 23 |
| Largest overlap (between genes) | rrnL–trnL1(tag) | nad1–nad4l |

**Table S6**. Start codons across the 13 PCGs

| **Start codon** *B. erythrostoma* | **Count** |
| --- | --- |
| ATG | 7 |
| ATT | 3 |
| ATA | 2 |
| TTG | 1 |
| **Start codon** *P. broderipii* | **Count** |
| ATG | 8 |
| ATT | 3 |
| TTG | 2 |

**Table S7**. PCGs annotated with incomplete stop codons (completed by polyadenylation)

| ***B. erythrostoma*** | ***P. broderipii*** |
| --- | --- |
| None detected | nad3, cox3, nad2 |

**Table S8**. Comparison of mitochondrial gene annotations obtained by manual curation (MITOS2-based) and MitoFinder for the complete mitogenome of *Plectostylus broderipii*.

| **Gene** | **Feature** | **MITOS2 + manual curation** | **MitoFinder** | **Difference (bp)** | **Strand** | **Result** |
| --- | --- | --- | --- | --- | --- | --- |
| COX2 | CDS | 1–660 | 1–660 | 0 | + | Identical |
| tRNA-Tyr | tRNA | 667–735 | 672–729 | −11 | + | Boundary refinement |
| tRNA-Trp | tRNA | 730–797 | 730–797 | 0 | + | Identical |
| tRNA-Gly | tRNA | 798–863 | 802–858 | −9 | + | Boundary refinement |
| tRNA-His | tRNA | 859–925 | 859–925 | 0 | + | Identical |
| tRNA-Gln | tRNA | 930–985 | 926–989 | +8 | − | Boundary refinement |
| tRNA-Leu1 | tRNA | 988–1050 | 990–1049 | −3 | − | Boundary refinement |
| ATP8 | CDS | 1080–1235 | 1080–1235 | 0 | − | Identical |
| tRNA-Asn | tRNA | 1287–1354 | 1287–1354 | 0 | − | Identical |
| ATP6 | CDS | 1377–2030 | 1377–2030 | 0 | − | Identical |
| tRNA-Arg | tRNA | 2096–2159 | 2096–2159 | 0 | − | Identical |
| tRNA-Glu | tRNA | 2157–2219 | 2157–2219 | 0 | − | Identical |
| rrnS | rRNA | 2220–2957 | 2220–2957 | 0 | − | Identical |
| tRNA-Met | tRNA | 2960–3026 | 2960–3026 | 0 | − | Identical |
| ND3 | CDS | 3027–3345 | 3028–3345 | −1 | − | Boundary refinement |
| tRNA-Ser2 | tRNA | 3386–3441 | 3386–3441 | 0 | − | Identical |
| tRNA-Ser1 | tRNA | 3440–3498 | 3440–3498 | 0 | + | Identical |
| ND4 | CDS | 3499–4812 | 3499–4812 | 0 | + | Identical |
| tRNA-Thr | tRNA | 4836–4903 | 4836–4903 | 0 | − | Identical |
| COX3 | CDS | 4904–5726 | 4905–5726 | −1 | − | Boundary refinement |
| tRNA-Ile | tRNA | 5917–5982 | 5916–5983 | +2 | + | Boundary refinement |
| ND2 | CDS | 5984–6903 | 5984–6901 | −2 | + | Boundary refinement |
| tRNA-Lys | tRNA | 6906–6972 | 6906–6972 | 0 | + | Identical |
| COX1 | CDS | 7343–8878 | 7343–8878 | 0 | + | Identical |
| tRNA-Val | tRNA | 8960–9023 | 8960–9023 | 0 | + | Identical |
| rrnL | rRNA | 9022–10086 | 9022–10086 | 0 | + | Identical |
| tRNA-Leu2 | tRNA | 10087–10154 | 10087–10154 | 0 | + | Identical |
| tRNA-Pro | tRNA | 10152–10214 | 10155–10211 | −6 | + | Boundary refinement |
| tRNA-Ala | tRNA | 10212–10275 | 10212–10275 | 0 | + | Identical |
| ND6 | CDS | 10322–10795 | 10322–10795 | 0 | + | Identical |
| ND5 | CDS | 10788–12455 | 10788–12455 | 0 | + | Identical |
| ND1 | CDS | 12448–13371 | 12448–13371 | 0 | + | Identical |
| ND4L | CDS | 13349–13648 | 13349–13648 | 0 | + | Identical |
| CYTB | CDS | 13650–14768 | 13650–14768 | 0 | + | Identical |
| tRNA-Asp | tRNA | 14778–14842 | 14778–14842 | 0 | + | Identical |
| tRNA-Cys | tRNA | 14846–14910 | 14846–14910 | 0 | + | Identical |
| tRNA-Phe | tRNA | 14912–14977 | 14912–14977 | 0 | + | Identical |

Note: Coordinates obtained after manual curation of the MITOS2 annotation were compared with an independent annotation generated using MitoFinder. All protein-coding genes, rRNA genes and gene orientations were recovered consistently by both methods. Differences were restricted to minor boundary adjustments (1–11 bp), mainly affecting tRNA genes and three protein-coding genes (ND2, ND3 and COX3).

**Table S9**. Comparison of mitochondrial gene annotations obtained by manual curation (MITOS2-based) and MitoFinder for the nearly complete mitochondrial genome of *Bostryx erythrostoma*.

| **Gene** | **Feature** | **MITOS2 + manual curation** | **MitoFinder** | **Difference (bp)** | **Strand** | **Result** |
| --- | --- | --- | --- | --- | --- | --- |
| COX1 | CDS | 1–1527 | 1–1527 | 0 | − | Identical |
| tRNA-Lys | tRNA | 1528–1586 | 1529–1583 | −4 | − | Boundary refinement |
| ND2 | CDS | 1593–2510 | 1593–2510 | 0 | − | Identical |
| tRNA-Ile | tRNA | 2518–2585 | 2518–2585 | 0 | − | Identical |
| COX3 | CDS | 2628–3446 | 2628–3446 | 0 | + | Identical |
| tRNA-Thr | tRNA | 3448–3518 | 3448–3518 | 0 | + | Identical |
| ND4 | CDS | 3510–4805 | 3510–4805 | 0 | − | Identical |
| tRNA-Ser1 | tRNA | 4847–4907 | 4909–4966 | −3 | − | Boundary refinement |
| tRNA-Ser2 | tRNA | 4897–4981 | 4847–4907 | −27 | + | Boundary refinement |
| ND3 | CDS | 4982–5326 | 4982–5326 | 0 | + | Identical |
| tRNA-Met | tRNA | 5327–5389 | 5327–5389 | 0 | + | Identical |
| rrnS | rRNA | 5401–6145 | 5401–6145 | 0 | + | Identical |
| tRNA-Glu | tRNA | 6140–6205 | 6140–6205 | 0 | + | Identical |
| tRNA-Arg | tRNA | 6207–6270 | 6207–6270 | 0 | + | Identical |
| ATP6 | CDS | 6273–6926 | 6273–6926 | 0 | + | Identical |
| tRNA-Asn | tRNA | 6943–7008 | 6943–7008 | 0 | + | Identical |
| ATP8 | CDS | 7043–7174 | 7043–7174 | 0 | + | Identical |
| tRNA-Leu1 | tRNA | 7187–7255 | 7187–7255 | 0 | + | Identical |
| tRNA-Gln | tRNA | 7267–7330 | 7267–7330 | 0 | + | Identical |
| tRNA-His | tRNA | 7329–7390 | 7329–7390 | 0 | − | Identical |
| tRNA-Gly | tRNA | 7387–7452 | 7391–7448 | −8 | − | Boundary refinement |
| tRNA-Trp | tRNA | 7453–7516 | 7453–7516 | 0 | − | Identical |
| tRNA-Tyr | tRNA | 7510–7580 | 7517–7575 | −12 | − | Boundary refinement |
| COX2 | CDS | 7581–8246 | 7581–8246 | 0 | − | Identical |
| tRNA-Phe | tRNA | 8250–8315 | 8250–8315 | 0 | − | Identical |
| tRNA-Cys | tRNA | 8345–8404 | 8342–8407 | +6 | − | Boundary refinement |
| tRNA-Asp | tRNA | 8422–8485 | 8422–8485 | 0 | − | Identical |
| CYTB | CDS | 8507–9626 | 8508–9626 | −1 | − | Boundary refinement |
| ND4L | CDS | 9712–10017 | 9712–10017 | 0 | − | Identical |
| ND1 | CDS | 10028–10933 | 10028–10933 | 0 | − | Identical |
| ND5 | CDS | 10915–12582 | 10915–12582 | 0 | − | Identical |
| ND6 | CDS | 12578–13033 | 12578–13033 | 0 | − | Identical |
| tRNA-Ala | tRNA | 13081–13145 | 13081–13145 | 0 | − | Identical |
| tRNA-Pro | tRNA | 13147–13209 | 13147–13209 | 0 | − | Identical |
| tRNA-Val | tRNA | 14169–14230 | 14169–14230 | 0 | − | Identical |
| tRNA-Leu2 | tRNA | 14334–14399 | 14334–14399 | 0 | − | Identical |
| rrnL | rRNA | 14374–15495 | 14374–15495 | 0 | − | Identical |

Note: Coordinates obtained after manual curation of the MITOS2 annotation were compared with an independent annotation generated using MitoFinder. All 37 mitochondrial genes (13 protein-coding genes, 22 tRNAs and 2 rRNAs) were consistently recovered by both methods. Differences were limited to minor boundary refinements (1–27 bp), primarily affecting tRNA genes, and one protein-coding gene (CYTB), while gene order and strand orientation remained identical.

**Table S10**. Comparison of the mitochondrial gene order in the COX1–ND6 region among representative Stylommatophora mitogenomes. Gene orders were oriented according to the transcriptional direction of COX1.

| **Species** | **Accession** | **COX1 strand** | **Gene order (COX1 → ND6)** | **Pattern** |
| --- | --- | --- | --- | --- |
| ***Bostryx erythrostoma*** | PX617854 | − | **COX1 → rrnL → trnL1 → trnV → trnP → trnA → ND6** | **Bostryx-like alternative** |
| ***Bostryx sp.*** | PV475493 | + | **COX1 → rrnL → trnL1 → trnV → trnP → trnA → ND6** | **Bostryx-like alternative** |
| ***Plectostylus broderipii*** | PX977766 | + | **COX1 → trnV → rrnL → trnL1 → trnP → trnA → ND6** | **Typical Stylommatophora-like** |
| *Camaena cicatricosa* | NC_025511 | + | COX1 → trnV → rrnL → trnL1 → trnP → trnA → ND6 | Typical Stylommatophora-like |
| *Dolicheulota formosensis* | NC_027493 | + | COX1 → trnV → rrnL → trnL1 → trnP → trnA → ND6 | Typical Stylommatophora-like |
| *Naesiotus nux* | NC_028553 | + | COX1 → trnV → rrnL → trnL1 → ND4L → trnP → trnA → ND6 | Other arrangement |
| *Aegista aubryana* | NC_029419 | + | COX1 → trnV → rrnL → trnL1 → trnP → trnA → ND6 | Typical Stylommatophora-like |
| *Deroceras reticulatum* | NC_035495 | + | COX1 → trnV → rrnL → trnL1 → trnP → trnA → ND6 | Typical Stylommatophora-like |
| *Microceramus pontificus* | NC_036381 | + | COX1 → trnV → rrnL → trnL1 → trnP → trnA → ND6 | Typical Stylommatophora-like |
| *Oreohelix idahoensis* | NC_043790 | + | COX1 → trnV → rrnL → trnL1 → trnP → trnA → ND6 | Typical Stylommatophora-like |
| *Ryssota otaheitana* | NC_044784 | + | COX1 → trnV → rrnL → trnL1 → trnP → trnA → ND6 | Typical Stylommatophora-like |
| *Cerion coloni* | NC_063566 | + | COX1 → trnA → trnP → trnV → rrnL → trnL1 → ND6 | Other arrangement |
| *Succinea arundinetorum* | NC_068814 | + | COX1 → trnV → rrnL → trnP → trnL1 → trnA → ND6 | Other arrangement |
| *Monacha cartusiana* | NC_072058 | + | COX1 → trnV → rrnL → trnL1 → trnP → trnA → ND6 | Typical Stylommatophora-like |
| *Arion flagellus* | NC_073101 | + | COX1 → trnV → rrnL → trnL1 → trnP → trnA → ND6 | Typical Stylommatophora-like |
| *Rabdotus mooreanus* | NC_082226 | + | COX1 → trnV → rrnL → trnA → trnP → trnL1 → ND6 | Other arrangement |
| *Eleutherocaulis alte* | PP716841 | + | COX1 → trnV → rrnL → trnL1 → trnW → trnF → trnL2 → ATP6 → trnR → trnQ → ATP8 → trnE → trnN → rrnS → trnM → trnT → COX3 → trnY → trnK → trnA → trnP → ND6 | Other arrangement |
| *Imerinia grandidieri* | JN619347 | + | COX1 → trnV → rrnL → trnL1 → trnA → trnP → ND6 | Other arrangement |
| *Lissachatina fulica* | NC_024601 | + | COX1 → trnV → rrnL → trnL1 → trnA → trnP → ND6 | Other arrangement |
|  |  |  |  |  |

Note: Gene orders were standardized according to the transcriptional orientation of COX1 to facilitate direct comparisons among taxa. Transfer RNA nomenclature (trnL1/trnL2 and trnS1/trnS2) was standardized across annotations.
