# Supplementary material for: Mitochondrial genomes and phylogenomic analysis of Plectostylus broderipii and Bostryx erythrostoma (Gastropoda, Stylommatophora, Orthalicoidea) from the Atacama Desert, Chile
Source: Zookeys. 2026 Sep 1;1291:1–18. doi: 10.3897/zookeys.1291.197983 (PMC13555134; doi:10.3897/zookeys.1291.197983)
Supplement: Supplementary file 2 [file zookeys-1291-001_article-197983__-s005.docx]

**Supplementary Material.**

**Manual curation of mitochondrial tRNA secondary structures**

Secondary structures of all mitochondrial transfer RNA (tRNA) genes were initially predicted using tRNAscan-SE v2.0, applying the mitochondrial invertebrate genetic code and covariance model-based prediction. The predicted anticodon identity and genomic coordinates were used as the initial annotation for each tRNA. Although covariance models accurately identify most mitochondrial tRNAs, several predictions produced atypical or poorly folded secondary structures, particularly for genes exhibiting reduced arms or lineage-specific structural variation. Therefore, all predicted structures were manually inspected individually rather than accepted automatically. Manual curation followed a comparative structural approach inspired by previous studies of metazoan mitochondrial tRNAs (e.g., Boore, 2005). For each tRNA, the following structural elements were evaluated: acceptor stem; DHU (D) stem and loop; anticodon stem and loop; variable loop; TΨC stem and loop.

The anticodon sequence and its genomic position were maintained unchanged throughout the curation process. Only stem boundaries were adjusted when necessary to maximize canonical Watson–Crick (A–U, G–C) and wobble (G–U) base pairing while preserving biologically realistic mitochondrial tRNA architecture. To guide manual refinement, homologous tRNAs from both gastropod mitogenomes analysed in this study, together with representative stylommatophoran mitochondrial genomes available in GenBank, were aligned using MAFFT v7 and, when required, covariance-model alignments generated with Infernal (cmalign). Comparative alignments were used to evaluate structural consistency among homologous tRNAs, under the expectation that homologous stem regions remain structurally conserved despite nucleotide substitutions. Candidate stems identified during automatic prediction were subsequently evaluated according to: number of canonical and wobble base pairs; stem length; loop length; conservation of anticodon position; consistency with homologous tRNAs from related taxa. No nucleotide substitutions were introduced during manual curation; only the placement of stem boundaries was adjusted where necessary to optimize biologically realistic base pairing.

Whenever multiple alternative folds were possible, the structure showing the greatest overall structural consistency across homologous sequences and the most canonical cloverleaf organization was selected. Particular attention was given to maintaining a complete acceptor stem and properly separated D, anticodon and T arms. Secondary structures were visualized using VARNA, and several structures were iteratively refined until a stable and biologically realistic cloverleaf conformation was obtained. Only a small number of tRNAs, principally members of the trnS family, retained atypical conformations after manual curation. These structures were preserved because they are consistent with the well-documented structural variability of mitochondrial serine tRNAs in Metazoa rather than representing annotation artefacts. The final curated structures presented in Supplementary Figures Sx–Sy therefore represent manually validated secondary structures supported by covariance-model prediction, comparative sequence alignment, and structural evaluation, rather than the direct output of a single prediction algorithm.

Summary of the curation workflow: tRNAscan-SE prediction → automatic secondary-structure prediction → manual inspection → comparative alignment (MAFFT + Infernal/cmalign, when required) → stem evaluation (canonical and wobble base pairing; anticodon conservation) → manual refinement in VARNA → final curated secondary structures.
